# Antigen Presenting Properties of a Myeloid Dendritic-Like Cell in Murine Spleen

**DOI:** 10.1371/journal.pone.0162358

**Published:** 2016-09-21

**Authors:** Ying-ying Hey, Helen C. O’Neill

**Affiliations:** 1 Research School of Biology, Australian National University, Canberra, ACT, Australia; 2 Clem Jones Research Centre for Regenerative Medicine, Bond University, Gold Coast, Queensland, Australia; INSERM, FRANCE

## Abstract

This paper distinguishes a rare subset of myeloid dendritic-like cells found in mouse spleen from conventional (c) dendritic cells (DC) in terms of phenotype, function and gene expression. These cells are tentatively named “L-DC” since they resemble dendritic-like cells produced in longterm cultures of spleen. L-DC can be distinguished on the basis of their unique phenotype as CD11b^hi^CD11c^lo^MHCII^-^CD43^+^Ly6C^-^Ly6G^-^Siglec-F^-^ cells. They demonstrate similar ability as cDC to uptake and retain complex antigens like mannan via mannose receptors, but much lower ability to endocytose and retain soluble antigen. While L-DC differ from cDC by their inability to activate CD4^+^ T cells, they are capable of antigen cross-presentation for activation of CD8^+^ T cells, although less effectively so than the cDC subsets. In terms of gene expression, CD8^-^ cDC and CD8^+^ cDC are quite distinct from L-DC. CD8^+^ cDC are distinguishable from the other two subsets by expression of *CD24a*, *Clec9a*, *Xcr1* and *Tlr11*, while CD8^-^ cDC are distinguished by expression of *Ccnd1* and *H-2Eb2*. L-DC are distinct from the two cDC subsets through upregulated expression of *Clec4a3*, *Emr4*, *Itgam*, *Csf1r* and *CD300ld*. The L-DC gene profile is quite distinct from that of cDC, confirming a myeloid cell type with distinct antigen presenting properties.

## Introduction

Dendritic cells (DC) play an important role in the immune system by acting as mediators between the innate and adaptive immune responses. Under steady-state conditions, DC process and present self antigen to T cells to maintain self-tolerance and prevent autoimmunity [1, 2]. DC become activated by danger signals and recruit other leukocytes to sites of infection [2]. Those that acquire infectious agents subsequently mount an antigen-specific T cell response against the pathogen [1, 2]. While several main DC lineages have been identified as professional antigen presenting cells (APC), increasingly novel APC are being identified in different tissues, each with specific functional capacity.

Both humans and mice contain multiple subsets of DC, characterized by distinct capacity for antigen uptake, processing and presentation leading to T cell activation [1, 3]. The DC lineage is therefore complex with distinct subtypes occupying different tissue locations, each with unique cell surface marker expression, migration potential, function in immunity and response to inflammatory states [1]. Conventional DC (cDC) are the main DC type in spleen, and comprise two subsets differing in CD8 expression. CD8^+^ cDC are phenotypically CD11c^hi^CD11b^-^CD8^+^MHCII^+^ cells, while CD8^-^ cDC differ in expression of CD11b and CD8 as CD11c^hi^CD11b^+^CD8^-^MHCII^+^ cells [4]. These two subsets are different in terms of cytokine production and capacity to take up antigen for cross-presentation [5]. CD8^+^ cDC are thought to maintain tolerance to self-antigens, consistent with their greater ability in cross-presentation [6, 7]. They are also the predominant source of IL-12, a cytokine which induces CD8^+^ T cell proliferation [1]. In contrast, CD8^-^ cDC have weaker cross-priming ability [6], but on stimulation with lipopolysaccharide (LPS), migrate from the resting location in marginal zone into T cell areas where they secrete inflammatory chemokines [6].

Professional antigen presenting cells process and present exogenous antigen as peptides in association with MHCII molecules as a prelude to activation of CD4^+^ T cells. However, cDC and in particular splenic CD8^+^ cDC, have been shown to have unique ability to process and cross-present exogenous antigen in association with MHCI molecules leading to activation of CD8^+^ T cells [8]. Cross-presentation can occur by two pathways [9]. The cytosolic pathway involves uptake of exogenous antigen by endosomes with subsequent release of antigen into the cytoplasm and degradation by proteasomes. Peptides are then transported into the endoplasmic reticulum by transporters associated with antigen processing (TAP) [9], loaded on to MHCI molecules, and then shuttled to the cell membrane. TAP and the MHCI loading complex have been described in phagosomes and endosomes [10, 11], so it is likely that loading of peptide onto MHCI can occur in the cytoplasm. By the vacuolar pathway, antigen processing occurs within endosomes containing lysosomes that break antigen down into peptides [9]. Loading of peptide on to MHCI occurs when vesicles carrying MHCI molecules fuse with peptide-rich endosomes. However, it is possible that DC can use both pathways under different conditions, and there is evidence in the literature which supports this possibility [10].

One limitation of studying the antigen presenting function of DC is the low number of these cells. One strategy has been to use *in vitro* culture methods to generate large numbers of DC for study. The first method generates monocyte-derived DC (mo-DC) from monocytes or myeloid progenitors using a cytokine cocktail comprising granulocyte macrophage colony-stimulating factor (GM-CSF), tumor necrosis factor (TNF)-α and interleukin (IL)-4 [12, 13]. The second method generates cDC and pDC from bone marrow-derived DC precursors under the influence of FMS-related tyrosine kinase 3 ligand (FLT3-L) [13–15]. Despite the ease of generating large numbers of cells by these *in vitro* methods, the DC derived are heterogeneous and activated, and not reflective of DC in the normal steady-state state [13]. An alternative strategy for isolation of DC for study is to use mice that constitutively express specific antigen, so reducing the need to pulse isolated cells with antigen *in vitro*, a method which leads to loss of cells with handling and washing. Use of these mice, reduces the number of APC needed to do experiments, as well as the preparation time required. For example, ACT-mOVA mice constitutively express membrane-bounded OVA under the actin promotor in all cells [16] so that APC can be directly isolated from spleen for testing antigen presenting function.

Recently we described a myeloid dendritic-like cell, namely L-DC, which is phenotypically distinct from known cDC and myeloid subsets in spleen [17]. These cells are also distinguishable from neutrophils and monocytes by their unique phenotype as CD11c^lo^CD11b^hi^CD8α^-^MHCII^-^Ly6C^-^Ly6G^-^Siglec-F^-^ cells [17]. These cells are not DC precursors or progenitors, nor do they arise from cultures supporting their growth with factors like Flt3L which supports cDC development [18, 19]. Previous studies have confirmed that L-DC are phenotypically very distinct from precursor DC in spleen [20–22]. They also arise *in vitro* in stromal co-cultures seeded with carefully sorted hematopoietic stem cells (HSC) and multipotential progenitors (MPP) but not from common dendritic progenitors (CDP) or precursor DC [19, 23]. Using *Ikaros* plastic mutant mice which have a defect which affects the self-renewal capacity of HSC, it was possible to identify the progenitor of L-DC as a self-renewing HSC [23]. Spleen has also been shown to contain HSC which give rise to L-DC when co-cultured above supportive stromal lines derived from spleen [24, 25]. A combination of studies therefore predict a myeloid dendritic-like cell type in spleen which arises endogenously from HSC in spleen.

The *in vivo* L-DC subset resembles a cell type which was previously defined in long-term stromal spleen cultures, and in co-cultures of hematopoietic progenitors over splenic stroma [17, 26, 27]. Early studies on *in vitro* generated L-DC also showed capacity to uptake dead tumour cells for generation of cytotoxic T cell responses reflecting cross-presenting capacity [28]. Recent studies on *in vitro* generated L-DC revealed capacity to take up external antigen and to activate CD8^+^ T cells through cross-priming, although cells were unable to activate CD4^+^ T cells [27]. Notably, these *in vitro* grown cells resemble dendritic as well as myeloid cells, on the basis of phenotype, but have ability to cross-prime CD8^+^ T cells [17], a property previously associated with cDC. In this study, a comparative study of the recently defined *in vivo* candidate L-DC subset [20] has been undertaken, comparing these cells with the well-defined cDC subsets in spleen. L-DC were sorted from spleen for direct comparison with subsets of CD8^+^ cDC and CD8^-^ cDC using phenotypic, functional and gene profiling methodology.

## Materials and Methods

### Animals

Animals were bred under specific pathogen-free conditions in the Biosciences Facility at the Australian National University (ANU), Canberra, ACT, Australia. Female mice were used at 6–8 weeks of age in all experiments. Mice were housed in a specific pathogen-free facility in individually ventilated cages using wood shavings as bedding in rooms regulated for light and ventilation at a constant temperature (19–24°C). Mice were supplied with sterile water and commercial grade rodent food pellets. Experimentation was conducted under protocol #A2013/11 approved by the Animal Experimentation Ethics Committee at ANU. Animals were euthanased using carbon dioxide asphyxiation to obtain tissues for cell isolation. The following mouse strains were used in experiments described here, with number shown in brackets: C57BL/6J (80), C57BL/6.Tg(TcraTcrb)1100Mjb (OT-I TCR-transgenic (tg) (anti-H-2K^b^/OVA_257-264_) (25), C56BL/6.SJL/J.OT-II.CD45.1 (OT-II TCR-tg (anti-IA^b^/OVA_323-339_) mice) (15) and C57BL/6-Tg(CAG-OVA)916Jen:WehiAnu (Act-mOVA) (115).

### Fractionation of cells

Dendritic and myeloid cells were isolated from dissociated whole spleen via red blood cell lysis followed by negative depletion of red blood cells and lymphocytes using magnetic bead separation and MACS^®^ technology (Miltenyi Biotec: Auburn, California, USA). T, B and red blood cell depletion was performed using specific antibody, i.e. 0.25μg biotinylated anti-Thy1.2 antibody/10^8^ cells (T cells), 0.25μg biotinylated anti-CD19 antibody/10^8^ cells (B cells) and 0.25μg biotinylated anti-Ter119 antibody/10^8^ cells (red blood cells) in 1mL fluorescence activated cell sorting (FACS) buffer (1% FSC, 0.1% sodium azide in Dulbecco's Modified Eagle Medium (DMEM)). Cells were washed and the supernatant discarded. They were then resuspended at 10^8^ cells/mL in MACS labelling buffer (2mM EDTA/0.5% Bovine Serum Albumin (BSA) in Phosphate-Buffered Saline (PBS)) and incubated on ice for 25 minutes. Cells were then washed twice, resuspended in MACS buffer (10^8^ cells/mL), followed by addition of 20μl of anti-biotin microbeads/10^8^ cells (Miltenyi) for 25 minutes on ice, washed once, and resuspended in 500μl of MACS labelling buffer prior to running cells through LS columns (Miltenyi) in a SuperMACS II Separation Unit (Miltenyi) to deplete T and B cells. After washing columns thrice, unbound cells were collected as flow-through cells.

CD8^+^ T cells were isolated from OT-I TCR-tg mice specific for ovalbumin (OVA_257-264_/H-2K^b^). Splenocytes were enriched for CD8^+^ T cells using MACS^®^ technology as described above, but with depletion of DC, granulocytes, myeloid, B cells and CD4^+^ T cells using specific antibodies, i.e. 0.25μg biotinylated anti-CD19 antibody/10^8^ cells (B cells), 0.25μg biotinylated anti-MHCII antibody/10^8^ cells (DC), 0.25μg biotinylated anti-Gr1 antibody/10^8^ cells (granulocytes and myeloid cells) and 0.25μg biotinylated anti-CD4 antibody/10^8^ cells. CD4^+^ T cells were prepared in a similar fashion from OT-II TCR-tg mice specific for ovalbumin (IA^b^/OVA_323-339_), by substituting anti-CD4 antibody with antibody to deplete CD8^+^ T cells (0.25μg biotinylated anti-CD8 antibody/10^8^ cells).

### Antibody staining

Antibody staining and flow cytometry were used to analyse cell surface marker expression as described previously [20]. Non-specific antibody binding via Fc receptors was blocked by incubating cells (≤10^6^) with anti-CD16/32 (FcBlock: Biolegend: San Diego, CA, USA) at 5μg/mL. Fluorochrome- or biotin-conjugated antibodies specific for CD11c (N418), CD11b (M1/70), CD8 (53–6.7), CD19 (1D3), CD43 (IBII), Ter119 (Ter119), Thy1.2 (30-H12), Siglec-F (E50-2440), Ly6C (HK1.4) and Ly6G (1A8) were purchased from Biolegend. Prior to flow cytometry, propidium iodide (PI: 1 μg/ml) (Sigma-Aldrich: St. Louis, MO, USA) was added to discriminate live and dead cells. Flow cytometry was performed on a BD LSRII flow cytometer (Becton Dickinson: Franklin Lakes, NJ, USA). Data collection involved forward scatter (FSC), side scatter (SSC) and multiple fluorescence channels detecting CFSE, fluorescein isothiocyanate (FITC), phycoerythrin (PE), PI, pacific blue (PB), Alexa-700, phycoerythrin-cyanine 7 (PE-Cy7), allophycocyanin (APC) and allophycocyanin-cyanine 7 (APC-Cy7). BD FACSDiva Software (Becton Dickinson) was used to acquire data. Data analysis involved post-acquisition gating using FlowJo software (Tree Star: Ashland, OR, USA).

### Cell culture

Cells were cultured in Dulbecco’s Modified Eagle Medium (DMEM) supplemented with 22.2mM D-glucose, 13μM folic acid, 27μM L-asparagine, 5.5mM L-arganine HCL, 10% heat inactivated Fetal Calf Serum (FCS) (JRH Biosciences: Lenexa, Kansas, USA), 10mM Hepes (JRH Biosciences), 2mM L-glutamine (JRH Biosciences), 17.1μM streptomycin (JCSMR), 100U penicillin and 50μM 2-mercaptoethanol (BDH Ltd.: Poole, England) per litre of medium. This is referred to as supplemented DMEM (sDMEM). Cells were maintained in 5% CO_2_ in air with 97% humidity at 37°C.

### Cell sorting

**Fig 1:**
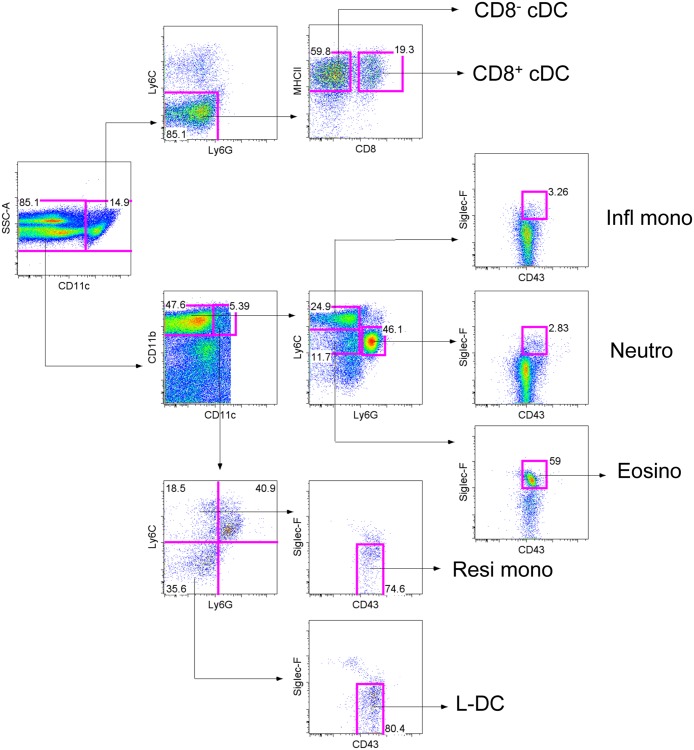
Phenotypic identification of cDC and L-DC in spleen. Representative flow cytometric analysis outlines the method used for delineation of subsets. This profile is reflective of multiple similar isolations involving individual mice. Splenocytes were prepared by red blood cells lysis followed by T and B cell depletion. Cells were then stained with antibodies specific for CD11b, CD11c, CD8, Ly6C, Ly6G, CD43 and Siglec-F. Prior to flow cytometry, cells were stained with propidium iodide (PI, 1μg/ml) to delineate live (PI^-^) cells. L-DC and myeloid cells were delineated on the basis of CD11b versus CD11c expression. Myeloid cells were gated as CD11b^hi^CD11c^-^ cells, and further delineated on the basis of Ly6C and Ly6G expression to reveal subsets of inflammatory monocytes (Infl mono) as Ly6C^hi^Ly6G^-^ CD43^+^Siglec-F^-^ cells, neutrophils (Neutro) as Ly6C^+^Ly6G^+^CD43^+^Siglec-F^-^ and eosinophils (Eosino) as Ly6C^+^Ly6G^-^CD43^+^Siglec-F^+^ cells. Resident monocytes (Resi mono) were gated as Ly6C^+^Ly6G^-^CD43^hi/+^Siglec-F^-^ cells, while L-DC were gated as Ly6C^-^Ly6G^-^CD43^+^Siglec-F^-^ cells. Conventional DC (cDC) were initially gated on the basis of side scatter (SSC) and CD11c expression. CD8^+^ cDC were gated as CD11b^-^CD8^+^Ly6C^-^Ly6G^-^ cells, while CD8^-^ cDC were gated as CD11b^+^CD8^-^Ly6C^-^Ly6G^-^ cells. Gates were set based on fluorescence minus one controls, and numbers in gates represent % specific binding.

Cells were stained with fluorochrome-labelled antibodies and subsets identified for sorting as described in Fig 1. All incubation and washing steps were performed in sodium azide-free FACS buffer. After a final wash prior to sorting, cells were filtered through a 70μm nylon cell strainer (Becton Dickinson) for removal of cell clumps. Sorted populations were collected in complete medium (sDMEM) for culture.

### Endocytosis assay

The capacity of cells to take up antigen was assessed in *in vivo* experiments. Ovalbumin conjugated to FITC (OVA-FITC) and mannan conjugated to FITC (mannan-FITC) were delivered intravenously (iv) to mice at 1mg/mouse at different times. Mice were sacrificed at the end of the timed study and their spleens harvested for analysis of cells. Splenocytes were RBC lysed and enriched via red blood cell, and T and B cell depletion as described above. After depletion, cells were collected for antibody staining and for flow cytometric analysis to delineate dendritic and myeloid subsets in spleen and to measure their antigen uptake.

### Activation of CD4^+^ and CD8^+^ T cells

The T cell activation capacity of DC was measured by their ability to induce antigen (OVA)-specific activation and proliferation of (1) CD4^+^ T cells isolated from anti-OVA specific OT-II TCR-tg mice, and of (2) CD8^+^ T cells from anti-OVA specific OT-I TCR-tg mice. Dendritic and myeloid cell subsets were sorted from spleens of transgenic Act-mOVA mice. Antigen presenting cells from these mice therefore express OVA antigen in the context of MHCI and MHCII after *in vivo* uptake and clearance of dead cells. Candidate APC subsets were sorted into sDMEM as described in Fig 1, and plated in diluting numbers prior to addition of purified T cells. Some APC were activated via addition of lipopolysaccharide (LPS, 10μg/mL) before addition of T cells.

T cells were purified from spleens of OT-I or OT-II mice and labeled with 5-(and 6-) carboxyfluorescein diacetate succinimidyl ester (CFSE: Molecular Probes: Eugene, Oregon, USA) in order to measure proliferation of cells following exposure to antigen. Proliferation was quantified flow cytometrically by the dilution of fluorescence with each cell division. In order to label T cells with CFSE, enriched cell populations were washed and resuspended at 10^7^cells/mL in CFSE labelling buffer (PBS/0.1%BSA). CFSE was added at a final concentration of 2.5μM and vortexed immediately upon addition to ensure uniform cell labelling. Cells were incubated at 37°C for 10 minutes followed by addition of 5 volumes of cold complete medium and incubation on ice for 5 minutes to quench labelling. Cells were pelleted (4°C, 5 minutes, 300g) and washed twice with complete medium. CFSE labelled T cells (10^5^) were added to diluting numbers of APC in a total volume of 200μL. After 4 days of co-culture, T cells were collected, stained with antibodies to clearly define the subset, and proliferation determined flow cytometrically by quantitation of CFSE staining in defined T cell subsets.

### Microarray analysis of gene expression

Splenic dendritic and myeloid subsets were sorted according to the gating procedure described in Fig 1. RNA was extracted from sorted subsets using an RNeasy mini kit (Qiagen: Clifton Hill. VIC, Australia) according to the manufacturer’s instructions. RNA was then labelled and hybridised to Mouse Gene 1.0ST genechips (Affymetrix: Santa Clara, CA, USA) by Dr Kaiman Peng (Biomolecular Resource Facility, ANU). The procedures used followed the Applause WT-Amp ST and WT-Amp Plus ST RNA Amplification Systems protocol published on the website of Nugen Technologies (San Carlos, CA, USA) (http://www.nugeninc.com/nugen/index.cfm/products/apl/applause-rna-amplification-systems/). Amplification of cDNA was performed with the SPIA amplification kit developed by NuGEN Technologies. The cDNA samples were fragmented and labeled according to the FL-Ovation^™^ cDNA Biotin Module V2 protocol (NuGEN Technologies), followed by hybridisation onto genechips (Affymetrix), which were then washed and stained using the fluidics station (Affymetrix) prior to scanning and analysis using a GeneArray^®^ Scanner (Affymetrix).

Scanned images of genechips were processed using Partek (St. Louis, Missouri, USA). Data files were prepared containing probeset numbers, gene descriptions, signal values and p-values in text file format. Data files were subsequently exported into Excel (Microsoft: Redmond, WA, USA) for further processing. Cells sorted from two separate experiments were prepared as replicates and ANOVA used to do pairwise analysis. Microsoft Excel was used to build a dataset of gene expression based on signal value and p value, comparing gene expression between subsets in terms of fold change. Datasets of genes specifically expressed by individual subsets, and genes common to several subsets, were selected using set criteria of signal value and p value.

### Statistical analysis

Data have been presented as mean ± standard error for sample size n. Where a normal distribution could be assumed, the Students’ *t*-test was used to determine significance (p ≤ 0.05). For sample size n ≤ 5, where a normal distribution cannot be assumed, the Wilcoxon Rank Sum test was used to test significant (p ≤ 0.05).

## Results

### Phenotypic identification of myeloid and DC subsets in spleen

Based on previous identification studies, L-DC can be gated as a CD11b^hi^CD11c^lo^Ly6C^-^Ly6G^-^CD43^+^Siglec-F^-^ subset (Fig 1) [20], and reflect myeloid lineage cells based on high expression of CD11b. Lack of Ly6C, Ly6G and Siglec-F expression delineates L-DC from other known subsets of monocytes, neutrophils and eosinophils. Monocytes can be delineated into subsets of resident and inflammatory monocytes. Resident monocytes were gated as CD11b^hi^CD11c^lo^Ly6C^lo^Ly6G^-^CD43^+/hi^Siglec-F^-^ cells, while inflammatory monocytes were gated as CD11b^hi^CD11c^-^Ly6C^hi^Ly6G^-^CD43^+^Siglec-F^-^ cells. Neutrophils were gated as CD11b^hi^CD11c^-^Ly6C^+^Ly6G^+^CD43^+^Siglec-F^-^ cells, while eosinophils are defined as CD43^hi^Siglec-F^+^ subset of CD11b^hi^CD11c^-^Ly6C^+^Ly6G^-^ cells. Splenic cDC subsets were isolated based on accumulated flow cytometry data in the literature [1, 3, 9]. The commonly used gating strategy identifies two cDC subsets by their distinct MHCII and CD8 expression. They were therefore gated as CD11c^hi^Ly6C^-^Ly6G^-^ cells, and then further delineated as CD8^+^ cDC and CD8^-^ cDC on the basis of MHCII expression and differential CD8 expression (Fig 1).

### Comparison of the endocytic capacity of L-DC with cDC

**Fig 2:**
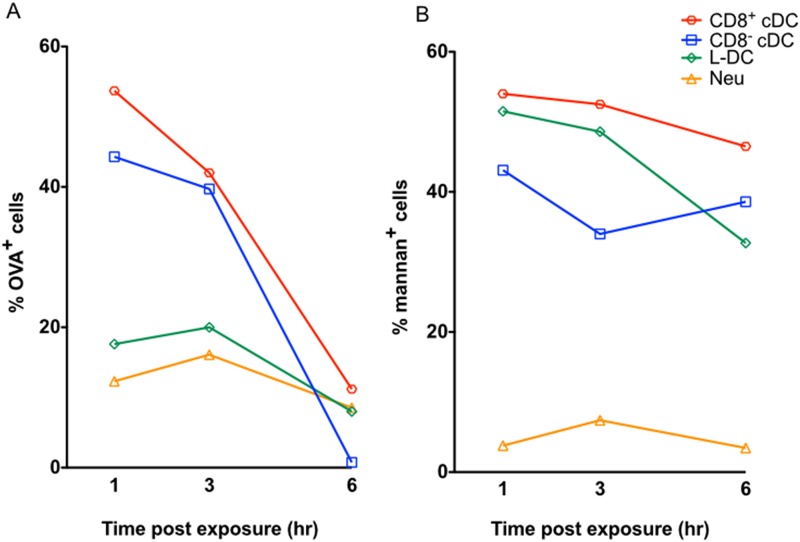
Comparison of endocytic ability of cDC subsets and L-DC subsets. The ability of cells to endocytose antigen was measured by uptake of FITC-conjugated ovalbumin (OVA-FITC) and FITC-conjugated mannan (mannan-FITC). C57BL/6J mice were given A) OVA-FITC (iv; 1mg per mouse), and B) mannan-FITC (iv; 0.5mg per mouse) at 1, 3 and 6 hours prior to euthanasia for spleen collection. Control mice were given PBS. Splenocytes were prepared by RBC lysis, and enrichment for dendritic and myeloid cells via T, B cell and red blood cell depletion. Cells were stained with antibodies and sorted to give L-DC, CD8^+^ cDC and CD8^-^ cDC subsets as shown in Fig 1. Uptake of antigen was assessed in terms of % FITC staining cells. Data are representative of 2 similar timed experiments.

Spleen is a lymphoid organ which also contains a red pulp compartment specialised for filtering blood and blood-borne antigens. It is expected therefore that splenic DC would be readily able to endocytose antigen. In this study, pinocytosis and receptor-mediated endocytosis of antigen were investigated for spleen DC subsets described in Fig 1. This involved infusion of labelled antigens into blood, with subsequent isolation of subsets to measure their uptake of antigen. When the soluble antigen OVA-FITC was given to mice by intravenous inoculation, approximately 50% of CD8^+^ cDC and CD8^-^ cDC demonstrated ability to endocytose and retain OVA over a 3-hour period, although this diminished to 10–20% after 6 hours (Fig 2A). For L-DC, ~20% of cells demonstrated ability to take up FITC-OVA, with ~10% of cells still retaining antigen after 6 hours (Fig 2A).

For receptor-mediated endocytosis, different cell types can have a unique combination of receptors on their cell membrane to aid uptake of antigen in various forms. Mannose receptor-mediated uptake of antigen by DC has been reported to play a role in cross-presentation for activation of CD8^+^ T cells [29, 30]. In order to determine if L-DC endocytose antigen via mannose receptors, mannan conjugated to FITC (mannan-FITC) was delivered intravenously to C57BL/6J mice and uptake monitored over time (Fig 2B). Both CD8^+^ cDC and CD8^-^ cDC demonstrated strong ability to take up mannan via mannose receptors. Over 50% of CD8^+^ cDC took up mannan-FITC and retained it for 6 hours, compared with ~40% of CD8^-^ cDC (Fig 2B). L-DC showed similar high ability to take up and retain mannan as did CD8^+^ cDC (Fig 2B). Neutrophils, as controls, did not endocytose mannan-FITC in this *in vivo* assay. In terms of comparing cells by their *in vivo* uptake of mannan, it must be remembered that results reflect both ability of cells for endocytosis as well as accessibility to blood-borne antigen.

### Ability of splenic DC subsets to activate CD4^+^ T cells

**Fig 3:**
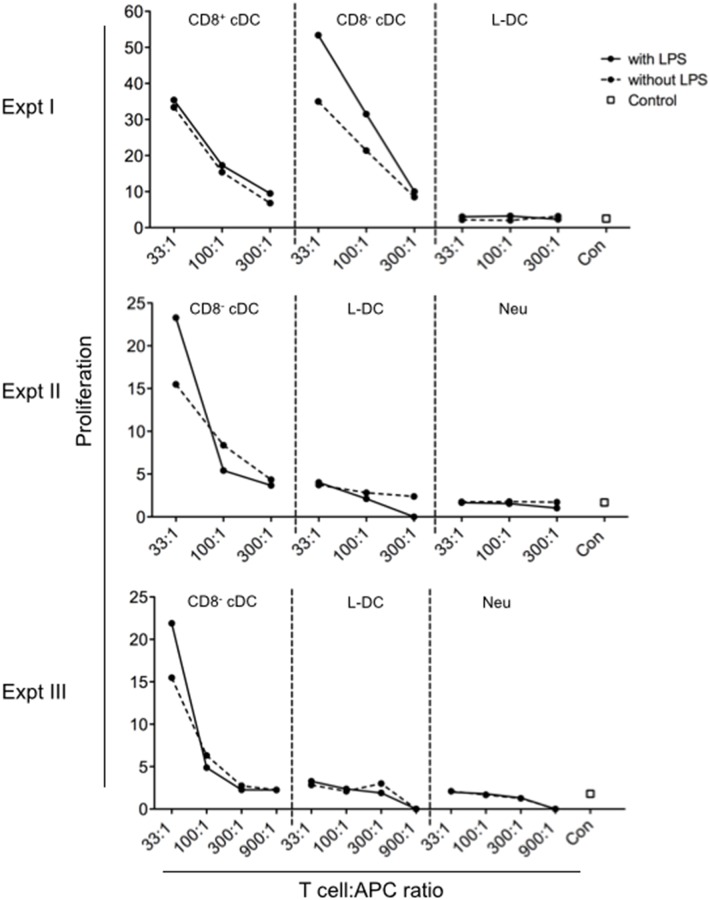
Activation of CD4^+^ T cells by splenic DC. Antigen presenting ability of DC subsets purified from spleens of Act-mOVA mice was compared. L-DC, CD8^+^ and CD8^-^ cDC, and neutrophils as a control, were sorted as described in Fig 1 following enrichment of splenocytes by depletion of red blood cells, and T and B lymphocytes using magnetic bead technology. Diluting numbers of APC were plated following treatment with and without LPS (10 μg/ml) for 2 hours. This was followed by addition of 10^5^ CFSE-labelled OT-II (TCR-tg) CD4^+^ T cells, purified from mouse spleen through depletion of B cells, CD8^+^ T cells, DC and myeloid cells using magnetic bead protocols. Cells were cultured at T cell:APC ratios of 33:1, 100:1, 300:1 and 1000:1 for 72 hours. CD4^+^ OT-II T cells were then gated as PI^-^Thy1.2^+^Vα2^+^CD8^-^ cells, and assessed flow cytometrically for CFSE dilution as an indicator of T cell proliferation. OT-II T cells cultured alone served as controls (con). Graphs show % proliferating OT-II cells. Three independent replicate experiments were conducted.

Previously it was shown that L-DC produced *in vitro* in either long-term cultures of spleen or in stromal co-cultures were unable to activate CD4^+^ T cells, a result consistent with their absence of MHCII expression on the cell surface [20, 22, 27]. These results contrasted with existing evidence that DC and other APC expressed MHCII and readily took up exogenous antigens for processing and presentation on MHCII for CD4^+^ T cell activation. CD8^-^ cDC are commonly known as the main APC for activation of CD4^+^ T cells [31]. In order to assess the functional capacity of the *in vivo* subset, L-DC along with CD8^+^ cDC and CD8^-^ cDC, were sorted from spleens of Act-mOVA mice and compared for capacity to induce activation and proliferation of CD4^+^ T cells isolated from OT-II TCR-tg mice. Both CD8^+^ cDC and CD8^-^ cDC induced proliferation of CD4^+^ T cells, with CD8^-^ cDC the stronger inducer (Fig 3) [32]]. Over three separate experiments, L-DC induced no response in line with the control subset of neutrophils. While addition of LPS gave ~3-fold increased response with CD8^-^ cDC, LPS had no effect on responses due to either CD8^+^ cDC, L-DC or neutrophils (Fig 3). This suggested that only CD8^-^ cDC expressed Toll-like receptor 4 which binds LPS. These results are consistent with the isolated *in vivo* L-DC subset being functionally distinct from cDC subsets. This also confirmed similarity between the classified *in vivo* subset of L-DC (Fig 1) [20] and *in vitro* generated L-DC.

### Cross-priming ability of DC subsets under normal and inflammatory states

**Fig 4:**
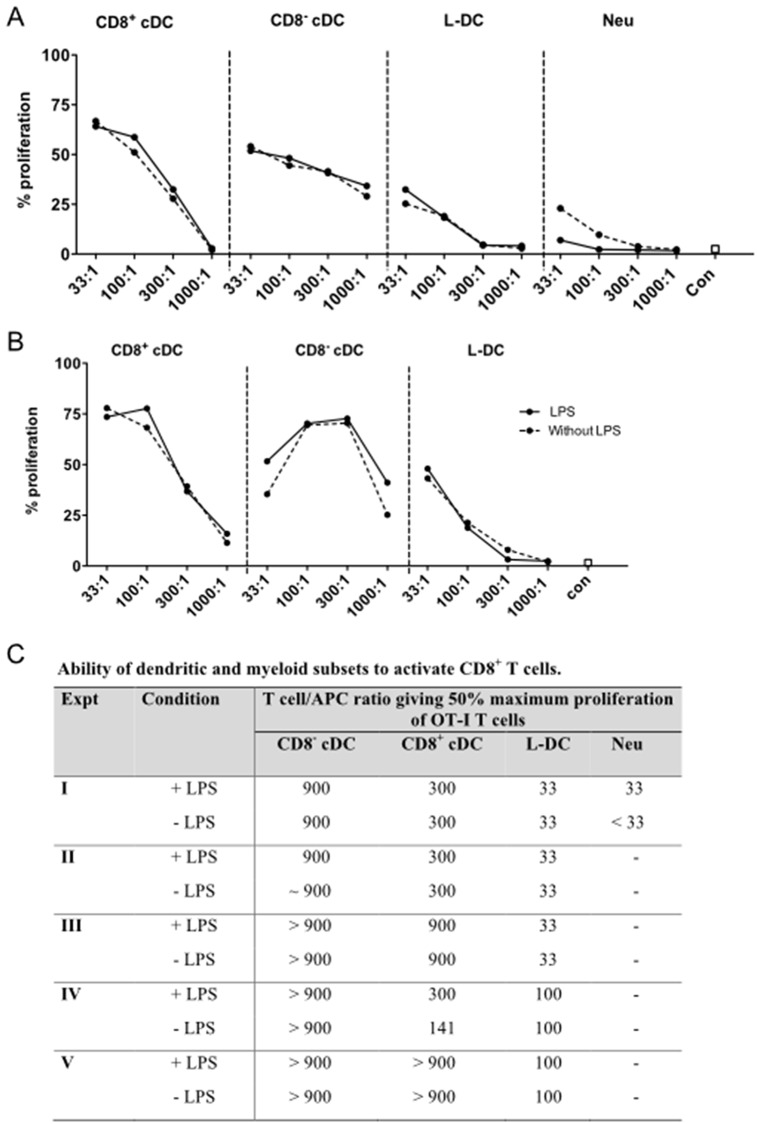
Cross-presentation ability of DC subsets. Cross-presentation of antigen was investigated for splenocytes harvested from Act-mOVA mice and prepared by red blood cell lysis and T/B cell depletion. Splenocyte subsets were stained and gated as described in Fig 1. Diluting numbers of DC were plated as APC followed by treatment with or without LPS (10 μg/ml) for 2 hours prior to the addition of 10^5^ CFSE-labelled OT-I (TCR-tg) CD8^+^ T cells, purified from OT-I mouse spleen through depletion of B cells, CD4^+^ T cells, DC and myeloid cells using magnetic bead protocols. A) CD8^+^ cDC, CD8^-^ cDC, L-DC and neutrophils, and B) CD8^+^ cDC, CD8^-^ cDC and L-DC were cocultured with APC in T cell:APC ratios of 33:1, 100:1, 300:1 and 1000:1, respectively. After 72 hours, CD8^+^ OT-I T cells were gated as PI^-^CD11b^-^Thy1.2^+^Vα2^+^ cells, and assessed flow cytometrically for CFSE dilution as an indicator of T cell proliferation. OT-I T cells cultured alone served as controls (con). Graphs show % proliferating OT-I cells. C) The T cell/APC ratio required to generate 50% maximum proliferation of OT-I cells was compared in the presence and absence of LPS across 5 replicate experiments.

Cross-presentation is a property which defines DC, although this property has been reported to be largely restricted to CD8^+^ cDC [32–35]. The splenic subsets of L-DC, CD8^+^ cDC and CD8^-^ cDC were isolated from ACT-mOVA mice and compared for ability to activate purified OTI (TCR-tg) CD8^+^ T cells (Fig 4). The assay was performed in the steady-state and in the presence of LPS as a potential inflammatory stimulus [36]. Across experiments I and II, shown graphically in Fig 4(A) and 4(B), both CD8^+^ cDC and CD8^-^ cDC showed strong ability to cross-prime CD8^+^ OT-I T cells. In this model system, CD8^-^ cDC were marginally stronger activators than CD8^+^ cDC. L-DC demonstrated much weaker ability requiring ~30-fold more L-DC over CD8^-^ cDC to induce an equivalent T cell proliferative response (Fig 4A, 4B and 4C). Neutrophils which can cross-present antigen only under certain inflammatory conditions [37] were used as a control in Experiment I showed almost no ability to induce proliferation in CD8^+^ T cells in the steady-state or following LPS activation (Fig 4A and 4C).

The ratio of T cell/APC required to induce 50% proliferation of CD8^+^ T cells was calculated in order to compare data across replicate experiments (Fig 3C). The collective data show that isolated CD8^-^ cDC from mACT-OVA mice demonstrate stronger ability to cross-prime than do CD8^+^ cDC. This result is contradictory to the literature which compares isolated normal CD8^+^ cDC and CD8^-^ cDC following *in vitro* pulsing with high concentrations of purified antigen [32, 38]. The combined data shows common trends, although some variability was noted between experiments. CD8^-^ cDC induced 50% maximum proliferation of CD8^+^ T cells at a ratio of 900 T cells/APC (Fig 4C). CD8^+^ cDC were the second best inducer of CD8^+^ T cell proliferation, requiring 300 T cells/APC to give half maximum T cell proliferation (Fig 4C). L-DC were required in higher number, and induced 50% maximum proliferation at ratios of 33 or 100 CD8^+^ T cells/L-DC (Fig 4C). In addition, the presence of LPS added into co-cultures did not improve the outcome of T cell activation across replicate experiments for all cell types tested (Fig 4C). The cross-presenting capacity of purified L-DC is not influenced by the addition of LPS, even though CD8^-^ cDC would appear to express the Toll-like Receptor 4 (TLR4) for LPS, since LPS-activated CD8^-^ cDC induced an increased CD4^+^ T cell activation response (Fig 3). This result compares with an earlier study to assess the cross-presenting capacity of a less pure population of L-DC which were isolated, cultured in vitro before pulsing with OVA as antigen [22]. That study revealed heightened response due to addition of LPS so that cells produced under those conditions were reflective of cells expressing TLR4 which binds LPS.

### The effect of cytochrome c on antigen presentation

**Fig 5:**
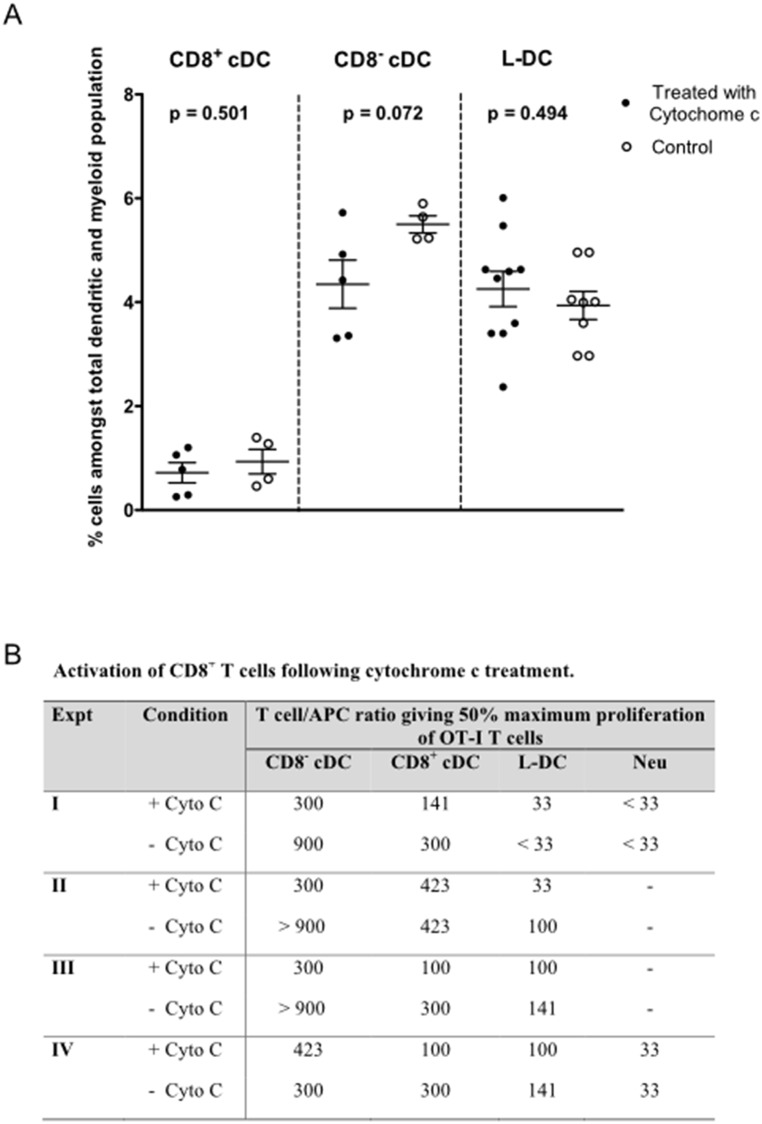
Effect of cytochrome c treatment on cross-presentation capacity. A) The *in vivo* killing effect of cytochrome c on APC was investigated in C57BL/6J mice. Cytochrome c (5mg/mouse) was delivered iv 6 hours prior to euthanasia for spleen collection. Control mice were given PBS. Splenocytes were prepared, stained and gated as described in Fig 1. Individual mice were analysed. Cell number is presented as % amongst the total dendritic and myeloid cell population. Mean and standard error (S.E.) are shown by cross bars. B) The effect of cytochrome c on cross-presentation of antigen was investigated using splenocytes harvested from Act-mOVA mice and prepared and sorted as described in Fig 1. Diluting numbers of APC were plated followed by treatment with or without cytochrome c (6 mg/ml) for 2 hours prior to the addition of 10^5^ CFSE-labelled OT-I (TCR-tg) CD8^+^ T cells, purified from OT-I mouse spleen through depletion of B cells, CD4^+^ T cells, DC and myeloid cells using magnetic bead protocols. Cells were cocultured with APC in T cell:APC ratios of 33:1, 100:1, 300:1 and 1000:1. After 72 hours, CD8^+^ OT-I T cells were gated as PI^-^CD11b^-^Thy1.2^+^Vα2^+^ cells, and assessed flow cytometrically for CFSE dilution as an indicator of proliferation. OT-I T cells alone served as controls (con). The T cell/APC ratio required to generate 50% maximum proliferation of OT-I cells was compared in the presence and absence of cytochrome c across 4 replicate experiments.

Cytochrome c treatment can be used effectively to induce apoptosis specifically in cells with cross-presenting ability [39]. It is endocytosed by APC in the same way as antigen, and released into the cytoplasm for cross-presentation via the cytoplasmic pathway. When cytochrome c enters the cytoplasm after uptake, it binds to apoptotic protease activating factor 1 (Apaf-1) to form an apoptosome which then induces a caspase cascade resulting in cell death [40]. Uptake of cytochrome c via endocytosis, and entry into the cytoplasm via cross-presentation, can lead to cell death [39]. After it was found that cytochrome c selectively killed CD8^+^ cDC, the mechanism for cross-presentation was thought to involve the cytosolic pathway [39]. L-DC were therefore compared with cDC subsets for sensitivity to cytochrome c treatment. Initially, cytochrome c was injected intravenously into mice, and changes in the *in vivo* representation of all DC subsets determined in spleen after 6 hours (Fig 5A). However, no difference was found in the size of the L-DC, CD8^+^ cDC, or CD8^-^ cDC populations in treated and untreated mice (Student’s t test: p ≤ 0.01). Further analysis of results for CD8^-^ cDC involved the Wilcoxon test since the variance of the treated and control populations was different. This test again showed no significant difference at p ≤ 0.05. The published *in vivo* effect of cytochrome c on numbers of CD8^+^ cDC were not reproduced here, although this could be attributed to reported variation in batches of cytochrome c [39].

Further studies then tested whether cytochrome c treatment had any effect on the cross-presenting capacity of splenic DC subsets measured *in vitro*. Sorted subsets of DC were treated with cytochrome c prior to culture with CFSE-labelled OT-I CD8^+^ T cells. Consistent with the literature, a drop in the cross-presenting ability of CD8^+^ cDC was observed when cells were treated *in vitro* with cytochrome c across multiple experiments (Fig 5B). This effect was reflected by a weaker T cell proliferative response. Similarly, reduced cross-presenting capacity was observed for CD8^-^ cDC (Fig 5B). Cytochrome c treatment of L-DC did reduce ability to cross-present antigen to CD8^+^ T cells in one experiment, but this was not a consistent result, and gave ~3-fold reduction in only one of four experiments (Fig 5B). In contrast, cytochrome c treatment resulted in a consistent three-fold increase in the number of CD8^-^ cDC and CD8^+^ cDC needed to give 50% maximum CD8^+^ T cell proliferation (Fig 5B). The cross-presenting capacity of CD8^+^ cDC and CD8^-^ cDC was clearly sensitive to cytochrome c treatment, but the case for L-DC was less certain, with variable, small effects. One interpretation is that the cytosolic pathway for cross-presentation of antigen is used by CD8^-^ cDC and CD8^+^ cDC, although it is not used by L-DC. As in Fig 4, neutrophils showed little ability to cross-prime CD8^+^ T cells (Fig 5B).

### Gene expression distinguishes L-DC from cDC subsets

**Fig 6:**
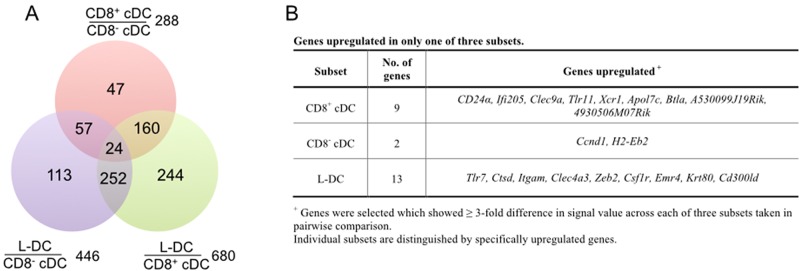
Differential gene expression. Subsets of CD8^+^ cDC, CD8^-^ cDC and L-DC were sorted from C57BL/6J mice using the antibody staining and gating strategy described in Fig 1. RNA was extracted from sorted subsets and converted to cDNA for label preparation, prior to hybridisation to Murine Gene ST1.0 genechips (Affymetrix). Data were analysed using Partek to give signal values and p values. ANOVA was employed to determine genes up- and down- regulated ≥ 3-fold in pairwise comparison. A) Number of genes upregulated ≥ 3-fold in one of two subsets assessed in pairwise comparison. B) Genes upregulated in only one of three subsets.

Gene profiling was conducted to identify potential genes and markers which distinguish L-DC from the cDC subsets, and to confirm that L-DC is a distinct cell type *in vivo*. Duplicate sorting experiments were conducted for mRNA preparation. Microarrays were employed to identify genes specifically upregulated in L-DC over the two cDC subsets. This involved the preparation of label from mRNA for hybridisation to Affymetrix Gene 1.0ST genechips. ANOVA analysis was used to make pairwise comparison of original values between subsets. Data were extracted for genes upregulated at least 3-fold in one subset only amongst the three, using a signal value of ≤50 to identify absence, but >150 for expression of genes. The number of genes specific to each dataset was represented in a Venn diagram as a measure of similarity or overlap between subsets (Fig 6A). Only 24 genes were differentially expressed across each of L-DC, CD8^+^ cDC and CD8^-^ cDC subsets (Fig 6A) and these are shown in Fig 6B.

The CD8^+^ cDC subset was found to be specifically marked by expression of *Xcr1*, *Ifi205*, *Tlr11*, *Btla*, *CD24α* and *Clec9a* (Fig 6B). These genes have previously been reported as specific markers of CD8^+^ cDC, and reflect their antigen presenting function [33, 41–45]. XCR1 binds to XCL1 secreted by T cells and aids the migration of DC to T cell areas within spleen, so promoting cross-priming of CD8^+^ T cells and development of a cytotoxic T cell response [44, 46]. Expression of CLEC9a is restricted to CD8^+^ cDC and plasmacytoid DC, and plays a specific role in the uptake of apoptotic cells via binding to the exposed actin filaments of damaged cells [47, 48]. Recently IFI205 was described as a receptor that regulates signaling via transcriptional regulation of the inflammasome adapter protein ASC [43]. BTLA has been described as an inhibitory receptor on DC that regulates T and B cell activation [49]. Genes upregulated by CD8^-^ cDC included only *Ccnd1* and *H2-Eb2*. CCND1 is involved in cell cycle progression [50], while H2-Eb2, also known as MHCII, is expressed by APC consistent with the capacity of CD8^-^ cDC to effectively activate CD4^+^ T cells [51].

L-DC showed specific upregulation of genes reflecting both myeloid and dendritic lineages (Fig 6B). *Clec4a3*, also known as *Dcir3*, encodes a C-type lectin expressed by DC [52]. *Emr4* encodes the epidermal growth factor receptor known to be expressed on CD8^-^ cDC, monocytes and some macrophages [53]. In addition, L-DC express high levels of *Zeb2* and *Krt80* involved in adhesion and migration [54, 55]. L-DC also express myeloid markers like *Itgam* and *Csf1r* [56, 57]. ITGAM, also known as CD11b, is a subunit of the heterodimeric integrin MAC-1, expressed by myeloid cells which mediates the inflammatory response by regulating adhesion and migration to sites of infection [58, 59], as well as participating in the phagocytosis of apoptotic cells [60]. Expression of CSF1R is consistent with myeloid lineage cells which respond to macrophage colony stimulating factor. It was however shown previously that L-DC do not express CD115 (CSFR1) as a cell surface marker, and are not dependent on CSF1 for their development in stromal co-cultures in contrast to cDC-like cells [19]. L-DC can also be distinguished by upregulated *Cd300ld*. This gene encodes a type I transmembrane protein with a short cytoplasmic tail and a charged transmembrane residue [61]. It is also expressed by granulocytes, monocytes, macrophages, monocyte-derived DC (mo-DC) and pDC, and interacts with the adaptor chain FcRγ to transmit an activation signal via LYN and SYK kinases [62].

### Identification of genes specifically expressed by L-DC

**Fig 7:**
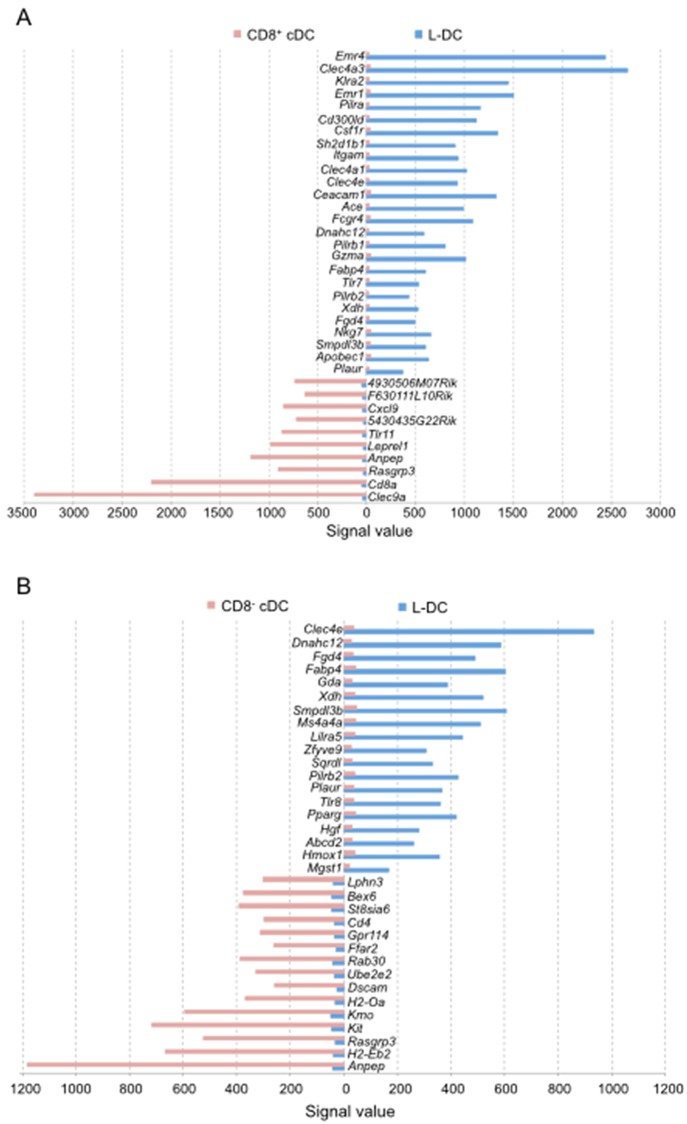
Specifically expressed genes which identify cDC or L-DC. ANOVA analysis was used to make pairwise comparison of gene expression between A) CD8^+^ cDC and L-DC, and B) CD8^-^ cDC and L-DC. Genes specifically expressed in one of two subsets were selected using the criteria of signal value in one subset ≤ 50, and signal value in the other ≥150. Comparison of CD8^+^ cDC and L-DC gave a dataset of 139 genes but the complete gene list is shown in supplementary data (S1 Fig). Only genes showing ≥ 15-fold difference in signal value are shown here. Comparison of CD8^-^ cDC and L-DC gave a dataset of 71 genes which are shown in S2 Fig. Only genes showing ≥ 8-fold difference in signal value are shown here.

Genes specifically expressed by the L-DC subset but not one or other of the cDC subsets were then identified in pairwise comparisons selecting genes with a signal value of ≤50 as not expressed, and those with a signal value ≥150, as expressed. In comparison with CD8^+^ cDC, L-DC were found to specifically express multiple genes related to myeloid cells (Fig 7A; S1 Fig). L-DC expressed *Itgam* (CD11b) [22], as well as *Klra2*, also known as *Ly49b*, which is expressed by monocytes, macrophages, NK cells and DC [63, 64]. Ly49B interacts with SHP-1, SHP-2 and SHIP to regulate signaling events [63]. L-DC also specifically expressed the *Pilrα*, *Pilrβ1* and *Pilrβ2* genes encoding proteins that regulate SHP signaling (Fig 7A). L-DC also specifically expressed genes related to macrophages including *Emr4*, *Emr1* and *Csf1r* (Fig 7A) [65–67], as well as *Nkg7* and *GzmaA* (Fig 7A), encoding granzymes involved in the induction of apoptosis.

L-DC also specifically expressed multiple genes related to DC although not CD8^+^ cDC, including *Clec4a3*, *Clec4a1*, *Clec4e*, *Ace*, *Fabp4* and *CD300ld* (Fig 7A). CLEC4 represents a family of transmembrane C type lectin receptors involved in diverse functions, including cell adhesion, signaling and inflammation [52]. L-DC expressed 2 isoforms of CLEC4A also described for CD8^-^ cDC [34, 68]. CLEC4E functions in antigen presentation, and initiation of inflammatory responses after cell death [69, 70]. The expression of CD300LD has been described for human myeloid lineage DC [71, 72]. L-DC also show expression of FABP4 described previously for human mo-DC, and involved in the production of IL-12 and TNF, as well as T cell priming [73]. Evidence presented in Fig 7 confirms that specific gene expression in L-DC over CD8^+^ cDC reveals that L-DC resemble both dendritic and myeloid lineage cells.

Genes specifically expressed by L-DC but not CD8^-^ cDC included *Clec4e*, *Pilrb2*, *Fabp4*, *Plaur*, *Dnahc12*, *Fgd4*, *Xdh* and *Smpdl3b*, which were also observed in the comparison of L-DC and CD8^+^ cDC (Fig 7A and 7B). In addition, L-DC specifically expressed *Ms4a4a*, *Zfyve9* and *Lilra5* which were not expressed by CD8^-^ cDC (Fig 7B). LILRA5 expression has been described for macrophages and induces production of proinflammatory cytokines and IL-10 in a rheumatoid arthritis model [74]. ZFYVE9 participates in TGF-β signalling by recruiting and interacting with SMAD2 and SMAD3 proteins [75]. MS4A4A is a novel protein which is also part of a signaling complex. L-DC specifically expressed multiple genes involved in signaling pathways which distinguish them from CD8^-^ cDC (Fig 7B; S2 Fig). Notably, L-DC did not express the *Zbtb46* gene recently shown to specify cDC development [76].

A final comparison of CD8^+^ cDC and CD8^-^ cDC also revealed many genes specific to each subset. This data is shown in the supplementary data (S3 Fig). In pairwise comparison, multiple known DC genes were revealed, so validating the experimentation. CD8^+^ cDC specifically expressed *Hepacam2* and *Itgae*, while CD8^-^ cDC specifically expressed *Emr1*, *Emr4*, *Clec4a4a*, *Clec4a1*, *Clec4a3*, *Gm9733*, *Dscam*, *Itgam* and *Apobec1* (S3 Fig). This analysis confirms that the sorted CD8^+^ and CD8^-^ cDC subsets were accurately identified and sorted in line with cDC subsets described in the literature.

## Discussion

In the past, DC subsets have been identified largely by their cell surface phenotype. However, this has led to confusion since DC can also express myeloid markers like CD11b and F4/80. A new nomenclature has been proposed whereby DC, monocytes and macrophages are primarily classified by their ontogeny and secondarily by their location, function and phenotype [77]. Splenic cDC are the best characterised murine DC and there have been extensive studies on their ontogeny, phenotype, function and gene profile [77]. In order to gain a better understanding of L-DC as a distinct *in vivo* subset, comparison studies were performed here with the CD8^+^ cDC and CD8^-^ cDC subsets in terms of their function and gene expression. In terms of gene profile, L-DC are quite distinct and mirror both dendritic and myeloid lineage cells. In terms of function, L-DC display capacity for receptor-mediated endocytosis of antigen and can activate CD8^+^ T cells through cross-presentation, although more weakly than splenic cDC. They do not however activate CD4^+^ T cells, which makes their classification as a DC less clear. Previously it was also shown that L-DC are distinguishable from splenic marginal zone and metaphillic macrophages since they do not express either SIGNR1 or MOMA-1 (data not shown).

When gene profiles of L-DC were compared with those of cDC subsets, L-DC were found to upregulate genes encoding both dendritic and myeloid markers (Fig 6B). By comparison with CD8^+^ cDC, L-DC specifically expressed myeloid markers like *Emr4*, *Clec4a3*, *Emr1*, *CD300ld*, *Csf1r* and *Itgam* (Fig 7A). However, when compared with CD8^-^ cDC, L-DC specifically expressed multiple genes involved in signaling pathways clearly distinct from CD8^-^ cDC (Fig 7B). Hence, the L-DC gene profile appears to suggest a myeloid dendritic-like cell distinct from both CD8^-^ cDC and CD8^+^ cDC.

The relationship between antigen uptake capacity and cross-presentation capacity has been studied here using specifically sorted cDC subsets and L-DC characterised in spleen [20]. L-DC show only receptor-mediated uptake of antigen which is also consistent with their capacity to cross-present antigen for activation of CD8^+^ T cells. Inability to pinocytose soluble antigen is also consistent with their inability to activate CD4^+^ T cells. There have been multiple reports suggesting that when APC take up antigen, the route of uptake can determine the processing and presentation pathway used for T cell activation [29, 30, 78]. Burgdorf and coworkers demonstrated that soluble antigen uptake in DC via pinocytosis gives different outcomes in terms of T cell activation compared with mannose receptor-mediated uptake [29, 30, 78]. They showed that uptake of antigen via the mannose receptor leads exclusively to activation of CD8^+^ T cells as evidence of cross-presentation, while uptake via pinocytosis leads to the activation of CD4^+^ T cells. However, their study did not specifically test cDC, since they isolated only CD11c^+^ splenocytes via density centrifugation and microbead technology, rather than by cell sorting. Here it is shown that splenic cDC and L-DC have distinct capacities to capture and retain antigen (Fig 2). CD8^+^ cDC and CD8^-^ cDC can be distinguished from other DC and myeloid subsets by their high endocytic ability, their strong cross-priming capacity, and their ability to activate both CD4^+^ and CD8^+^ T cells. In comparison with cDC, L-DC demonstrated strong mannose receptor-mediated uptake of antigen, but weak capacity to endocytose soluble antigen (Fig 2). While a good activator of CD8^+^ T cells, L-DC did not activate CD4^+^ T cells (Fig 3). Previously, a functional mannose receptor was considered to be a marker of cross-presentation ability for splenic APC subsets [30, 79]. Here, it was found that L-DC have similar capacity for mannose receptor-mediated uptake as do CD8^+^ cDC, but a 10-fold lower ability to cross-present antigen (Fig 4C).

Conventional DC are rare cells representing about one percent of spleen leukocytes, while L-DC are present in even lower frequency [80]. The use of purified subsets of DC in *in vitro* assays has been limited by low recovery of cells. To overcome these limitations, ACT-mOVA transgenic mice were employed as a source of APC [16]. In these mice, cross-presentation of antigen occurs *in vivo*, when APC endocytose dead cells and cell debris. However, since the ACT-mOVA mouse model expresses high levels of cell-associated OVA protein, it is also possible that some OVA might enter the endogenous antigen processing pathway. For example, defective OVA could be tagged for ubiquitin destruction in the cytoplasm, entering proteasomes, and transported into the endoplasmic reticulum. For this reason, neutrophils, that do not normally cross-present antigen in wild type mice, were used to assess background levels of cross-presentation due to the endogenous pathway in ACT-mOVA mice. Neutrophils from ACT-mOVA mice, in the absence of LPS stimulation, showed no ability to cross-prime CD8^+^ T cells, indicating that OVA processing was not occurring via the cytoplasmic pathway in a cell type which lacks antigen presenting capacity. However, LPS treated neutrophils showed very weak ability to prime CD8^+^ T cells, consistent with reports that bone marrow and peritoneal neutrophils can prime CD8^+^ T cells under inflammatory conditions [37].

Using the ACT-mOVA mouse model, and *in vitro* analysis of function, CD8^-^ cDC were found to have the strongest capacity to prime CD8^+^ T cells, despite having nearly the same ability to take up antigen via mannose receptors as CD8^+^ cDC and L-DC (Figs 6 and 7). CD8^+^ cDC had cross-presenting capacity up to 3-fold weaker than that of CD8^-^ cDC (Figs 2 and 4). L-DC showed 30-fold weaker capacity to cross prime CD8^+^ T cells than did CD8^-^ cDC, while still giving a measurable response (Figs 2 and 4). The hypothesis that antigen uptake via the mannose receptor is an indicator of cross-presenting ability is not consistent with data presented here. Others have also questioned that theory, and Segura and coworkers demonstrated that gene knock-out of aminopeptidase IRAP encoding an enzyme essential for proteolytic breakdown of antigen in the endosome did not affect the cross-presenting ability of CD8^+^ cDC [81]. Similarly, knockdown of mannose receptors in CD8^+^ cDC did not impact ability to cross-present antigen [81, 82]. However, gene knockdown of IRAP and mannose receptor did adversely impact ability of *ex vivo* isolated monocyte-derived DC (mo-DC) to cross-prime [82]. One explanation for these discrepant findings could lie in the purity of DC populations under study. Another could be that DC have multiple receptors on the cell membrane which participate in the endocytosis of antigen, also consistent with their ability to clear apoptotic cells from the environment [83]. Thus, for CD8^+^ cDC at least, receptors other than the mannose receptor might contribute to the uptake of antigens, and these may contribute to cross-priming of CD8^+^ T cells in mannose receptor and IRAP knockout mice.

## Conclusions

In summary, transcriptome analysis showed clear differences in gene expression between the splenic subsets of L-DC, CD8^+^ cDC and CD8^-^ cDC, with L-DC expressing genes indicative of both dendritic and myeloid cell lineages. While CD8^+^ cDC and CD8^-^ cDC are similarly strong activators of CD8^+^ T cells through cross-presentation, L-DC are relatively weak activators. Since cross-presentation has been considered a hallmark characteristic of DC which distinguishes them from other myeloid subsets, L-DC would appear to reflect a distinct myeloid dendritic-like cell.

## Supporting Information

S1 Fig Genes specifically expressed in L-DC or CD8^+^ cDC. (PDF)

S2 Fig Genes specifically expressed in L-DC or CD8^-^ cDC. (PDF)

S3 Fig Genes specifically expressed by CD8^+^ cDC and CD8^+^ cDC. (PDF)
