# Supplementary material for: Antigen Presenting Properties of a Myeloid Dendritic-Like Cell in Murine Spleen
Source: PLoS One. 2016 Sep 21;11(9):e0162358. doi: 10.1371/journal.pone.0162358 (PMC5031434; doi:10.1371/journal.pone.0162358)
Supplement: S1 Fig — (PDF) [file pone.0162358.s001.pdf]

S1 Fig. Genes specifically expressed between L-DC and $CD8^+$ cDC.

Gene Symbol	Fold-Change (L-DC vs. $CD8^-$ cD) $^+$	L-DC		$CD8^+$ cDC	
		Mean*	Standard Error	Mean*	Standard Error
Zeb2	14.837	548.052	67.119	36.668	0.693
Ifitm6	14.407	646.978	112.504	44.228	0.583
Gm9733	14.262	508.325	16.090	35.626	0.282
Gzmb	13.854	451.871	24.728	33.097	5.901
Ms4a4a	13.817	511.336	80.421	36.838	4.618
Atp1a3	13.665	528.196	5.192	38.733	2.487
L1cam	13.556	448.746	22.570	33.109	1.794
Gda	12.573	385.173	7.133	30.667	1.525
Sqrdl	12.326	328.967	23.265	27.184	5.500
Pltp	12.081	485.865	58.788	39.922	0.244
Lrp1	11.975	375.113	16.648	31.303	0.711
Lilra5	11.826	442.285	10.181	37.485	2.662
Ncr1	11.540	393.951	22.797	34.091	0.864
C3	11.306	425.896	13.786	37.653	0.302
Pla2g7	11.213	510.647	32.392	45.452	0.237
Tlr8	11.029	361.373	32.602	32.676	1.666
Abcd2	10.848	260.523	7.352	24.036	1.217
Nupr1	10.478	481.366	36.486	45.904	2.918
Hgf	10.139	278.901	5.863	27.518	0.972
Ikzf3	9.856	290.713	35.031	29.362	2.181
Fam46a	9.784	378.418	16.911	38.660	1.309
Krt80	9.635	423.455	0.465	43.950	0.235
Sepp1	9.454	326.572	47.610	34.184	0.839
Gm5150	9.449	367.228	82.481	37.895	1.292
A630033H20Rik	9.332	320.050	30.498	34.168	1.417
Irak3	9.060	264.136	8.938	29.259	2.663
Pparg	8.974	421.427	17.184	46.938	1.276
Abca9	8.492	234.244	11.094	27.566	0.790
Klra8	8.341	236.170	3.963	28.324	0.895
Fcgr3	8.200	300.711	28.094	36.512	0.215
Zfyve9	8.196	308.588	0.476	37.663	0.892
Fam55d	8.157	251.301	1.203	30.864	1.892
Arhgef3	7.919	387.354	66.081	48.211	1.141
Mgst1	7.917	166.937	11.405	21.039	0.326
S1pr5	7.904	303.395	4.288	38.477	2.729
Plxnb2	7.784	287.536	21.735	36.948	2.883
Mdm1	7.713	260.629	0.366	33.795	0.634
Ccl9	7.643	328.078	28.768	42.763	0.557
Gimap4	7.342	289.814	26.903	39.306	0.373
Svil	7.290	256.716	10.133	35.245	2.054
Trem3	7.262	357.258	13.473	49.163	0.101
Cd300lb	7.242	218.947	2.567	30.237	0.582
Ccdc125	6.898	197.052	2.197	28.629	1.895
Trem1	6.609	211.974	11.743	32.028	0.462
Gstm1	6.574	212.010	26.095	32.115	2.644
Rhoq	6.467	256.344	4.777	39.630	0.193
Tmem26	6.400	244.988	24.831	38.142	2.108
Xylt1	6.398	273.414	24.012	42.585	1.186
Rtp4	6.380	197.046	9.031	30.881	1.326
Pros1	6.351	179.988	24.135	28.108	1.130
Apoc2	6.340	240.037	11.321	37.820	0.295
Hp	6.313	182.405	43.577	28.123	1.913

Tmem71	6.311	151.394	20.686	23.769	0.444
Fpr1	6.308	169.278	2.425	26.835	0.312
Thbd	6.226	211.722	25.817	33.902	3.149
S100a6	6.167	219.687	25.302	35.415	1.409
Cd84	6.158	228.672	10.700	37.366	4.491
Rbpms	6.026	288.010	12.871	48.001	4.910
Tbc1d2b	5.971	191.380	0.900	32.084	1.493
Csf3r	5.969	163.212	1.153	27.365	1.077
Map3k15	5.930	247.350	7.442	41.866	3.836
B4galt5	5.804	270.397	21.621	46.762	5.476
Cyp4f18	5.798	195.922	7.464	33.830	2.110
Abi3	5.691	188.720	13.844	33.098	1.328
Gcnt2	5.659	201.374	9.994	35.548	0.638
Ms4a6d	5.547	158.618	7.103	28.609	1.567
Rap1gap2	5.470	212.381	9.855	38.794	0.906
6430548M08Rik	5.397	215.089	13.036	39.859	2.514
Pdlim1	5.343	244.698	2.334	45.881	2.751
Alox5	5.338	176.833	3.283	33.130	0.641
Ttc39a	-5.281	41.159	1.918	217.696	15.663
Amica1	-5.347	38.433	1.812	206.023	17.717
Trpm2	-5.403	44.730	0.361	244.203	35.127
Frmd4b	-5.486	43.374	2.417	237.666	6.732
Gca	-5.498	45.694	2.830	252.937	33.173
Fam149a	-5.810	27.369	1.392	159.397	13.597
Fnip2	-6.095	42.679	5.517	259.547	28.938
Actn1	-6.103	34.869	2.537	212.299	5.480
Met	-6.298	35.512	2.706	223.965	20.668
Cxcl16	-6.382	42.028	0.692	268.406	11.225
Ppef2	-6.640	32.921	0.067	218.863	10.681
Fndc7	-6.643	29.337	0.427	194.899	4.072
Nek6	-6.724	34.587	2.361	232.055	4.099
H2-Oa	-6.780	32.437	0.130	221.097	22.887
Cdon	-6.952	41.034	2.449	284.776	0.732
Rab30	-7.135	42.721	0.268	304.795	0.546
Clec1a	-7.266	29.651	0.328	215.436	2.336
Itgae	-7.569	29.068	0.032	221.400	24.737
Rnf144b	-7.821	33.829	4.037	264.515	31.007
Ece1	-7.880	47.285	0.344	374.364	36.515
Plekha5	-8.317	42.413	6.056	349.171	6.188
Fam40b	-8.470	30.717	1.708	262.130	35.061
Ciita	-8.991	43.440	2.971	391.061	33.038
Gatm	-9.527	32.792	2.301	311.982	14.869
Kmo	-10.016	47.450	3.216	474.173	1.057
Adam19	-10.264	40.698	1.667	418.164	25.395
Gcet2	-10.314	30.685	0.161	316.697	11.734
Pdia5	-10.391	34.510	2.207	360.117	40.376
Adam8	-10.538	45.131	0.709	476.002	21.794
Hepacam2	-11.851	23.649	1.339	280.052	11.621
Kit	-12.288	45.381	3.567	556.738	30.154
Cacna1e	-13.444	34.281	0.870	461.562	27.567
Ly75	-13.465	44.834	0.452	603.775	10.825
Tlr3	-14.975	30.232	1.223	452.356	2.728

$^{+}$ Genes were selected which showed between 5 to 15 fold change in signal value where one subset has signal value ≤ 50 and the comparison subset has a signal value ≥ 150, in either L-DC or CD8^{+} cDC assessed in pairwise comparison.

* Data represent mean of duplicate samples (n=2).
