# Supplementary material for: Antigen Presenting Properties of a Myeloid Dendritic-Like Cell in Murine Spleen
Source: PLoS One. 2016 Sep 21;11(9):e0162358. doi: 10.1371/journal.pone.0162358 (PMC5031434; doi:10.1371/journal.pone.0162358)
Supplement: S2 Fig — (PDF) [file pone.0162358.s002.pdf]

S2 Fig. Genes specifically expressed between L-DC and CD8⁻ cDC.

Gene Symbol	Fold-Change (L-DC vs. CD8⁻ cDC)+	L-DC		CD8⁻ cDC	
		Mean*	Standard Error	Mean*	Standard Error
Gm10673	7.387	272.591	56.193	36.702	7.355
Fcgr3	7.119	300.711	40.598	42.165	3.019
Irak3	6.566	264.136	22.644	40.625	5.823
Map3k15	6.537	247.350	9.595	37.823	0.491
Hp	6.532	182.405	36.805	27.146	1.271
Tmem26	6.344	244.988	38.620	38.677	4.480
Mdm1	6.070	260.629	15.696	43.076	3.419
Apoc2	6.045	240.037	10.652	39.798	3.288
Fpr1	5.936	169.278	11.785	28.583	2.002
Thbd	5.904	211.722	22.901	35.616	1.195
Xylt1	5.803	273.414	32.386	46.949	1.091
Cd300lb	5.783	218.947	26.248	38.000	3.319
Svil	5.765	256.716	16.560	44.520	1.425
Trem1	5.757	211.974	20.345	36.820	2.085
Pros1	5.710	179.988	20.266	31.242	0.695
Ccdc125	5.227	197.052	15.631	37.781	2.532
Gstm1	5.092	212.010	16.306	41.796	6.312
Lrrc16a	-5.055	35.121	73.789	177.984	16.044
Ptpn3	-5.076	30.921	2.082	157.520	13.583
Ptpn3	-5.165	47.759	7.073	246.474	10.867
Dennd3	-5.320	37.311	4.596	199.139	16.767
Ctnnd2	-5.372	33.902	1.754	182.360	12.929
Flnb	-5.546	48.463	6.019	270.441	29.982
Bub1	-5.601	34.466	5.957	195.833	36.781
Card11	-5.850	36.686	2.685	214.530	18.356
Tns1	-6.049	32.084	2.670	194.485	12.789
Mical3	-6.091	46.421	5.960	282.759	12.329
Clec9a	-6.333	45.057	3.686	285.108	36.356
Gpm6b	-6.405	34.712	5.485	223.304	21.383
Tbc1d4	-6.438	42.156	3.074	272.840	28.020
Siglecg	-6.908	49.420	4.720	338.777	11.596
St8sia1	-7.381	29.334	8.984	217.773	23.448
B4galt6	-7.506	44.314	6.252	333.803	32.710
Cdon	-7.539	41.034	2.337	309.066	12.666
Ciita	-7.679	43.440	2.430	332.968	10.191
Ptpn3	-7.950	33.400	4.627	266.634	30.051
1300002K09Rik	-7.991	41.162	3.429	329.781	24.192

+ Genes were selected which showed between 5 to 8 fold change in signal value where one subset has signal value ≤ 50 and the comparison subset has a signal value ≥ 150, in either L-DC or CD8⁻ cDC assessed in pairwise comparison.

* Data represent mean of duplicate samples (n=2).
