# Supplementary material for: Antigen Presenting Properties of a Myeloid Dendritic-Like Cell in Murine Spleen
Source: PLoS One. 2016 Sep 21;11(9):e0162358. doi: 10.1371/journal.pone.0162358 (PMC5031434; doi:10.1371/journal.pone.0162358)

S3 Fig. Genes specifically expressed by CD8+ cDC or CD8- cDC.

ANOVA analysis was used to make pairwise comparison of gene expression between the CD8+ cDC and CD8- cDC subsets. Specifically expressed genes were selected using the criteria of signal value in one subset ≤ 50, and signal value in the other ≥ 150. This gave a dataset of 35 genes.

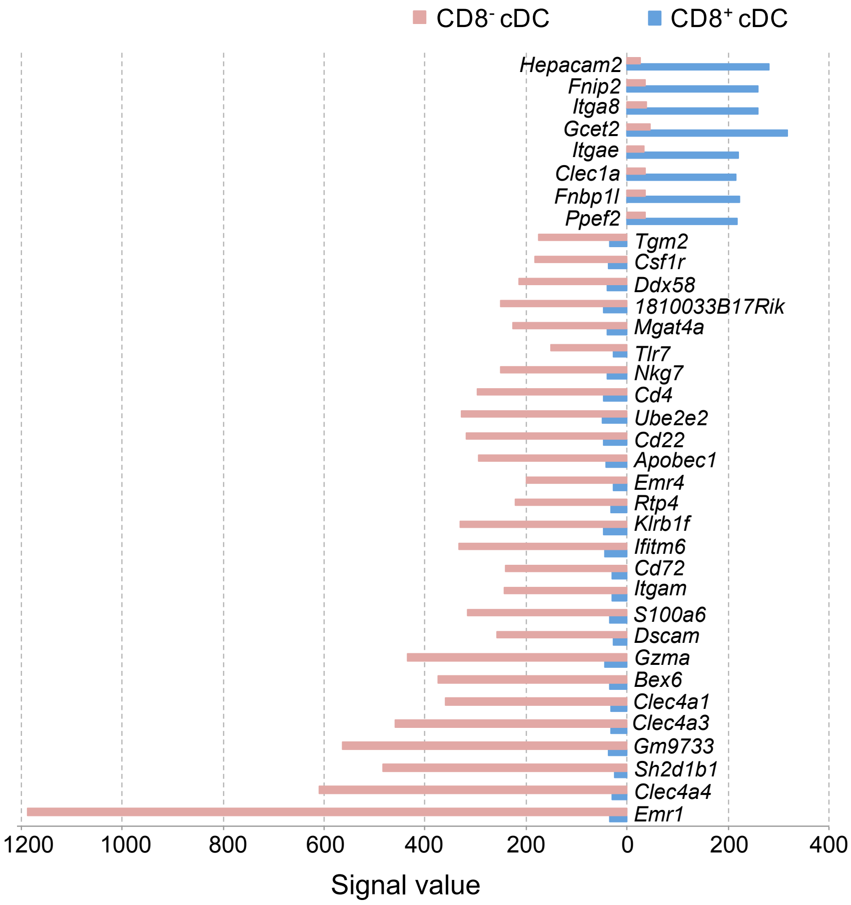

Supplement: S3 Fig — (PDF) [file pone.0162358.s003.pdf]
